# Improving the quality of surgical morbidity and mortality conference using a standardized reporting and assessment tool: a validation study from a large academic medical center in the United States

**DOI:** 10.1186/s13037-025-00433-3

**Published:** 2025-04-04

**Authors:** Sarah M. Dermody, Marc C. Thorne, Robert J. Morrison

**Affiliations:** 1 Department of Otolaryngology-Head & Neck Surgery, University of Michigan, Ann Arbor, MI USA; 2 1500 E Medical Center Dr, 1904, 48109 Taubman, Ann Arbor, MI USA

**Keywords:** Morbidity and mortality, Quality improvement, Resident education, Process improvement, Safety events

## Abstract

**Background:**

The purpose of this study is two-fold: (1) Improve the quality of Morbidity and Mortality conferences by developing a standardized presentation template and assessment tool; (2) Assess the intervention impact by comparing pre- and post-intervention data.

**Methods:**

A pre-post study was conducted at a tertiary care academic medical center between January 2022– January 2023. A standardized presentation template was created and a short assessment tool was developed to evaluate the quality of presentations on eight domains. We hypothesized that development of this template would significantly improve the quality of M&M conferences. Pre- and post-intervention data were compared using the Kruskal-Wallis test to evaluate for significant differences. Effect sizes for each domain were assessed by Cohen’s d.

**Results:**

A total of 127 pre-intervention responses and 61 post-intervention responses were received over a six-month period. Statistically significant increases in post-intervention scores were noted in nearly all presentation domains, including clarity of case selection rationale, nature of the safety event, circumstances leading to the safety event, contributing factors, understanding of the safety event, and anticipated benefits to patient outcomes (*p* < 0.05). The effect sizes ranged from medium for rationale for case selection to small for the identification of corrective actions.

**Conclusions:**

The introduction of a standardized, guided template improved the quality of Morbidity and Mortality presentations, with medium effect sizes and statistically significant increases in nearly all surveyed domains. A ceiling effect in the overall assessment score was noted as presentations prior to the intervention were rated highly. Standardization of case selection and presentations can promote alignment of the Quality Improvement Morbidity and Mortality workflow with broader-scope initiatives, departmentally and institutionally.

## Background

Due to the longstanding role of the Morbidity and Mortality (M&M) conference in improving patient safety, evidence guiding best practices for this Patient Safety and Quality Improvement (PSQI) mechanism is of great interest to the surgical and medical community [1]. These conferences serve as an integral part of surgical departments nationwide, although the content and structure of case presentations can differ greatly between departments and even presenters [1–9]. M&M conferences can act as a nidus for generation of PSQI efforts, but the content presented during the discussion of a case as well as the principles guiding the conference are critical to achieving this goal [4, 10–12].

The inconsistency and ineffectiveness of the M&M conference is pervasive throughout the surgical community and has been acknowledged for decades [9, 13–15]. M&M conferences can be affected by recall biases and a reluctance to openly report circumstances that may lead to blame [1]. A recent review suggests that surveys can be designed to measure the effectiveness of M&M conferences [1]. Although several studies have piloted methods for assessing presentations and providing feedback, specific guidance on the structure and content of such presentations is lacking [16, 17]. The lack of a formal structure to assist in proper case analyses has been cited as an area in which improvement efforts should be focused [14, 18–19].

The most recent systematic review published in 2023 sought to analyze the currently available literature regarding the attributes of M&M conferences [4]. There is a paucity of literature on this topic, with less than 60 studies identified, most of which were judged to be of average quality [4]. Several studies have highlighted the importance of communication and feedback regarding progress from M&M conferences [20, 21]. Some groups have discussed implementation of a structured slide template [22] and others have emphasized the importance of concise case presentations [2, 3]. There are several reports that inclusion of faculty moderator can enhance educational value [3, 22–23]. After systematically reviewing the current literature, Beaulieu-Jones et al. suggest several factors that are essential to high-quality M&M conferences, including: (1) preparation and post-conference follow-up, (2) succinct case presentation and discussion of educational topics, (3) encouraging accountability and multi-stakholder discussion (Beaulieu-Jones). Our study targets all three of these factors.

The objectives of our study were two-fold: (1) to improve the quality of the University of Michigan Department of Otolaryngology– Head and Neck Surgery M&M presentations through the development of a standardized presentation template and assessment tool; and (2) to assess the impact of these quality improvement efforts by comparing pre- and post-intervention data over a six-month period. We hypothesize that formulation of a standardized presentation template will improve the quality of M&M conference as measured by pre- and post-survey analysis.

## Methods

We hypothesize that implementation of a standardized presentation template for surgical M&M conference will objectively improve the quality of presentations as measured by a reporting and assessment tool. This study was conducted at a large academic medical center in Ann Arbor, MI between January 2022– January 2023. The study was designed as a pre-post study using survey results as the main outcome measure for assessment of the intervention tool.

### Case submission and selection

Faculty and residents in the Department of Otolaryngology– Head and Neck Surgery at our tertiary care academic medical center were responsible for submitting cases for potential review through the M&M process. The criteria for case submission included all surgical complications, all complications of medical care, all deaths within 90 days of surgery, unscheduled readmissions within 30 days of surgery, unplanned return to the operating room within 30 days of surgery, other safety events involving patient harm, and other near-miss events that have the potential to result in patient harm. Cases were reviewed by department PSQI leadership (including the Associate Chair for Education and Quality and the Quality and Outcomes Director) and, when appropriate, the Professional Practice Evaluation Committee.

The selection of cases was made to address one or both service goals of the M&M conference mechanism, including: (1) Patient Safety Events (PSE) and/or (2) Educational Opportunity. PSEs were defined as events where deviation of generally accepted standard practice (as defined by the Health Performance Improvement LLC) has occurred or ambiguity in practice would be served by discussion of systems-level improvement [24] Educational Opportunities were defined as instances where discussion of known complications would be beneficial to educate participants on appropriate identification and management, methods of prevention, or disclosure. The presenter of selected cases was the resident or fellow most closely involved in the complication, safety event, or the patient’s care.

The guiding principles of the department M&M conference included the following: Errors must be accepted as system flaws, not character flaws; errors are seen as consequences, not causes; it is the process, not the individual, who failed; and it is the cause of the error, not the error itself, which leads to productive prevention strategies.

### Presentation template

A standardized presentation template for the department M&M conference was created to encourage presenters to consider a variety of domains important to discussion of complications and safety events. The goal of this presentation template was to ensure a consistent level of quality and content across presentations and provide structure for a ten-minute presentation followed by five minutes of moderator-led discussion. At our institution, three cases are typically presented at each one-hour monthly conference.

**Fig. 1:**
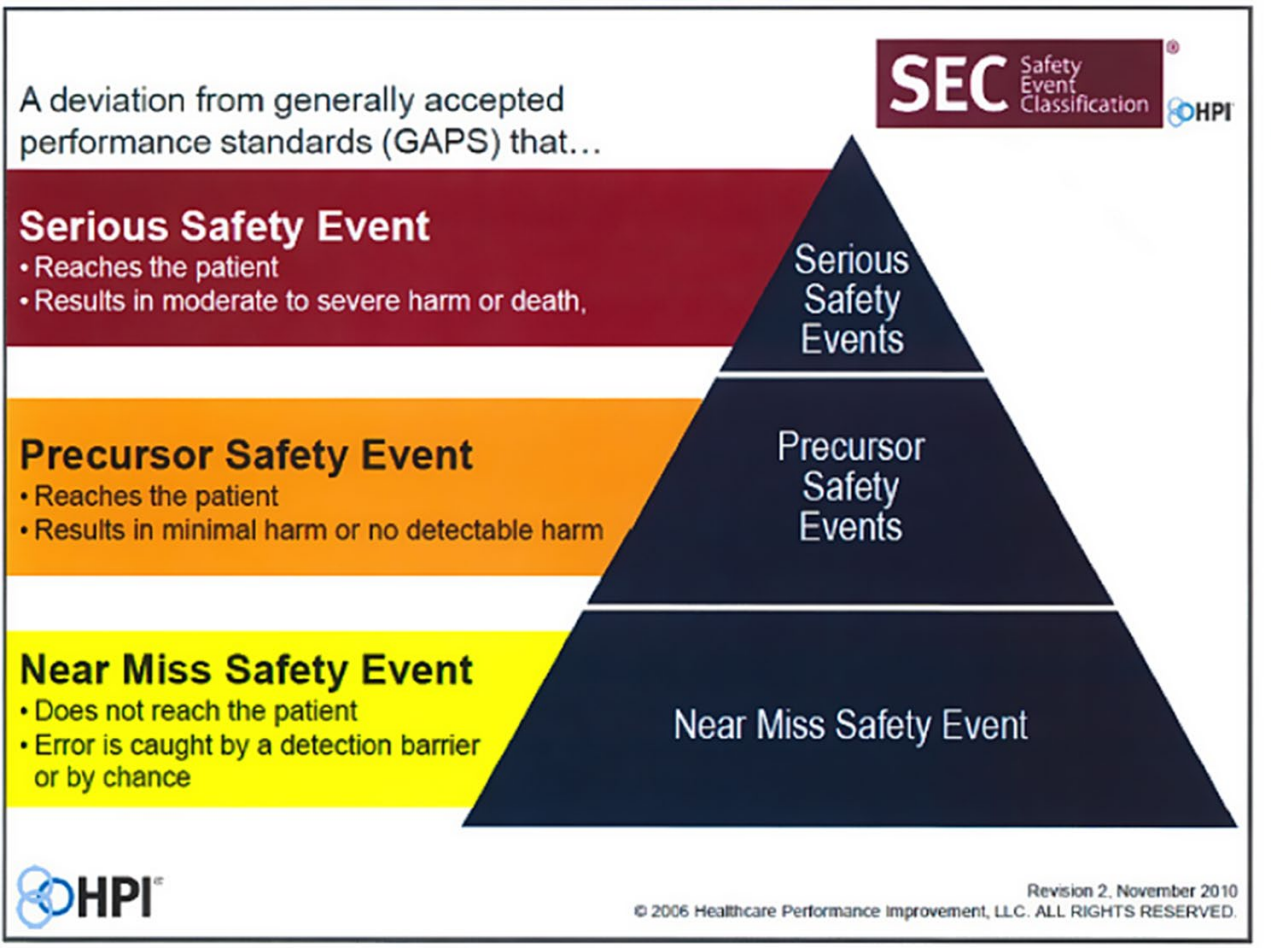
Safety Event Classification from Hillard et al

The presentation template included eight slides to optimize concise and clear presentations. After the title slide, presenters were prompted to insert a one-sentence summary of the clinical scenario (e.g. wrong-site surgery, nerve injury, incorrect medication) on the “Case Information” slide. This slide also included explicit rationale for case election as Patient Safety Event (PSE) or Educational Opportunity. For PSEs, presenters were then asked to classify the event as defined by the Healthcare Performance Improvement (HPI) Safety Event Classification framework (Fig. 1) [24].

The subsequent slide involved a “Case Review” in which the presenter provides a single slide to concisely summarize pertinent clinical history and/or an event story map. The next slide discussed “Potential Contributing Factors,” which include questions regarding potential cultural competency biases, cognitive biases, and behavior issues. The subsequent slide was “Apparent Cause Analysis” which was used to identify the cause(s) of deviation from the standard of care. The goal of this slide was to identify possible root causes of the event. One slide was dedicated to “Literature Review” in which the presenter can describe any literature related to establishing the standard of care and if available, prevention of apparent causes. The penultimate content slide was for “Summary and Action Items,” which mimics the Root Cause Analysis (RCA2) model for safety event review, where the presenter summarizes the event, apparent causes, and provides suggested actions for PSQI generation following the M&M conference [25]. The final slide was reserved for “Moderator Led Discussion.”

### Assessment tool

**Fig. 2:**
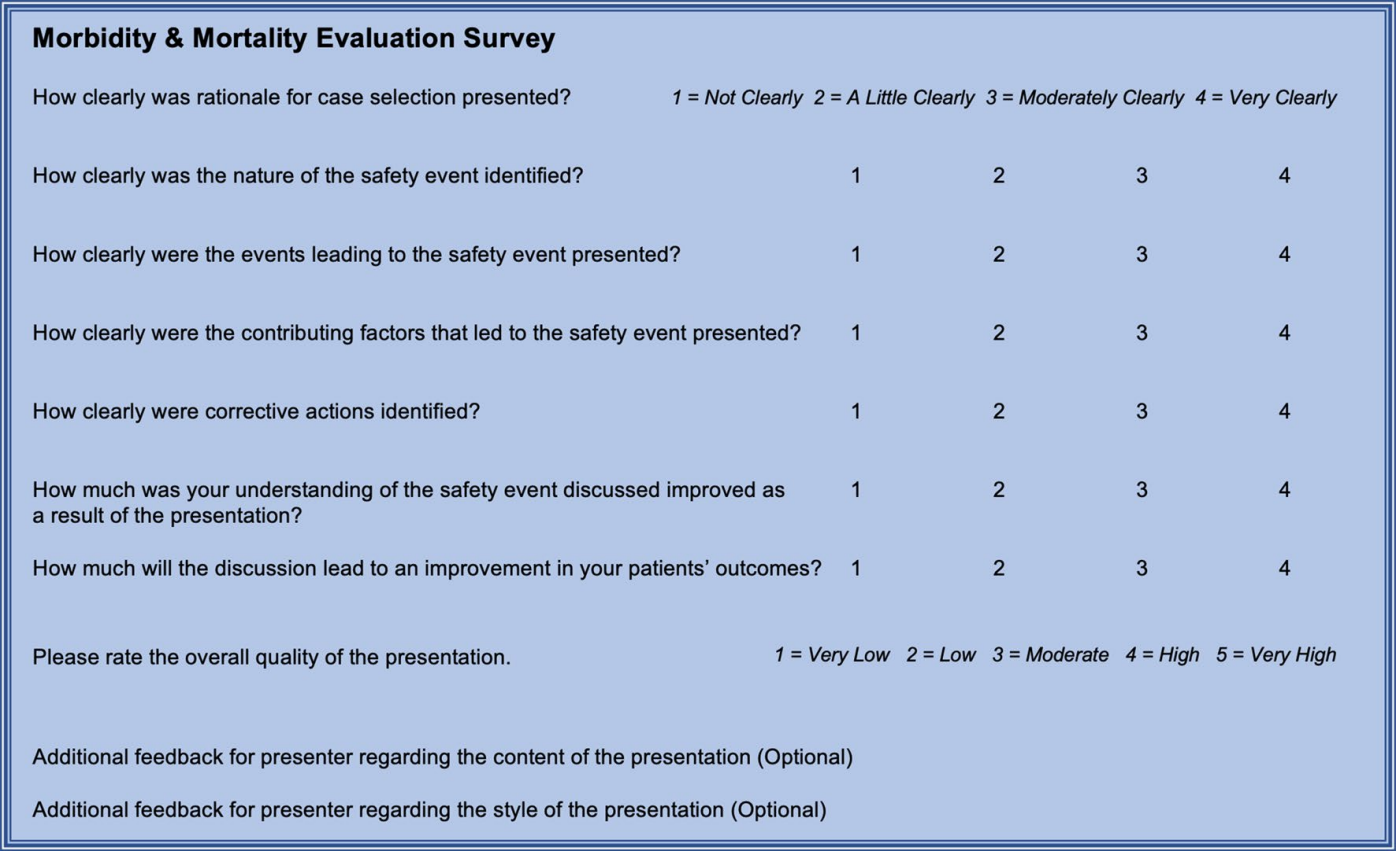
Assessment tool survey questions

A short assessment tool was developed to evaluate the quality and content of M&M conference presentations. Eight domains were assessed by the survey instrument: case selection rationale, nature of safety event, events leading to safety event, contributing factors, corrective actions, understanding of safety event, improvement in patient outcomes, and overall quality. The assessment tool was formatted via the Qualtrics^XM^ platform (Provo, UH) and distributed electronically to conference attendees for three monthly conferences prior to the introduction of the template and three monthly conferences following the introduction of the presentation template (Fig. 2).

### Statistical analyses

Pre- and post-intervention data were compared using *Stata* (College Station, TX, USA). Survey results for each of eight domains was stratified and compared in the pre-intervention surveys and post-intervention surveys. The Kruskal-Wallis test was used to evaluate for significant differences across the eight queried domains above by comparing the median value of each group. The effect sizes for each domain were assessed using Cohen’s d test. This study was deemed exempt from full committee review by the University of Michigan Institutional Review Board (HUM00066405).

## Results

A total of 127 conference attendee responses were received prior to the introduction of the standardized template. In the three-month period following template introduction, 61 survey responses were received. Statistically significant increases in post-intervention scores were noted in the following domains: case selection rationale, nature of the safety event, circumstances leading to the safety event, contributing factors, understanding of the safety event, and improvement in patient outcomes (*p* < 0.05). Effect sizes ranged from medium for rationale for case selection [Cohen’s d 0.63, 95% CI (0.32–0.95)] to small for identification of corrective actions [Cohen’s d 0.27, 95% CI (0.03–0.58)]. A ceiling effect in the overall score was noted as presentations prior to intervention were rated highly (4.5/5). Table 1 details the pre- and post-intervention effect sizes for each of the 8 domains assessed.

**Table 1:** Effect sizes of survey domains

Domain	Pre-intervention mean	Post-intervention mean	Effect Size	95% CI
Case selection rationale	3.55	3.92	0.64 (medium)	0.32–0.94
Nature of safety event	3.66	3.90	0.45 (medium)	0.15–0.76
Events leading to safety event	3.63	3.90	0.44 (medium)	0.13–0.75
Contributing factors	3.56	3.90	0.53 (medium)	0.22–0.84
Corrective actions	3.61	3.80	0.27 (small)	0.03–0.58
Understanding of safety event	3.35	3.61	0.35 (small)	0.04–0.66
Improvement in patient outcomes	2.88	3.78	0.53(medium)	0.22–0.84
Overall presentation quality	4.50	4.72	0.33 (medium)	0.02–0.64

## Discussion

Regular M&M conferences are a mandatory requirement for residency training programs nationwide according to the Accreditation Council for Graduate Medical Education (ACGME) [26]. Despite the recognition of the importance of reviewing M&M cases, the ACGME provides no guidelines on the structure, content, or format of such discussions [26]. In 2012, the Association for Surgical Education published the SBAR model as a valid tool to assess the quality of surgical M&M conference presentations [16]. This model provides a way to qualitatively and quantitatively assess and provide feedback for M&M conferences. Other surgical departments have piloted tools to provide formative feedback for surgical resident grand rounds presentations [17, 21, 27–29]. Standardization of patient safety event review workflows has been increasingly adopted amongst healthcare systems [25]. This work provides a starting point for improving resident M&M presentations. Our study builds on these concepts not only by utilizing an assessment tool but also by providing an explicit template for case presentations which may prove useful in this rapidly evolving area of research.

This study provides a replicable methodology for improving M&M conferences in surgical departments. The use of a standardized template provides clear expectations to ensure a consistent level of quality and content across all presentations. Through the assessment of pre- and post-intervention data, we demonstrated that our standardized template was effective in improving the quality of case presentations. Following our institution’s success, this presentation template can be broadly and easily implemented across other institutions across the country. Our assessment tool may also be broadly applicable as a feedback mechanism for presenters.

Mechanisms must exist to enact change to address root causes identified in M&M conferences. Translation of identified problems into concrete actions is essential for improving the effectiveness of M&M conferences [14]. The moderator-led discussion portion of a M&M case presentation provides an opportunity to incorporate salient points into a broader Quality Improvement structure that can then inspire specific PSQI interventions. By explicitly including a slide on suggested actions, we provide concrete steps to carry out such improvements.

Our long-term goal of M&M conference improvement is translation of conference findings into tangible actions. A database including all cases that met threshold for a safety event where system level intervention was recommended was created to track progress and outcomes. One illustrative example of enacted change involved a case in which a patient was not on venous thromboembolism (VTE) prophylaxis while admitted to the hospital, despite the provider placing such orders. In this specific case example, VTE orders were placed after surgery while the patient was still in the perioperative phase of care setting. With transfer of phase of care to inpatient, these orders would “fall off” as the patient was admitted to the floor. This was a known failure state which residents were familiar with and had developed workaround solutions for years. After M&M conference discussion and programmatic follow-through, an electronic medical record (EMR) wide system fix was deployed within two weeks. VTE prophylaxis orders are now automatically transitioned to new phases of care for all patients.

Limitations of our work include the single academic institution setting which may limit sample diversity and is subject to institutional biases. Single center studies are subject to temporal limitations as well, in which the study may only capture data points from a period of institutional leadership. The University of Michigan has a strong commitment to PSQI and therefore we do not anticipate that our work will change in the event of policy shifts. While our study involved a single academic institution, we believe that our template and assessment tool can be across surgical disciplines and academic institutions. The template and assessment tools can be adapted to fit the goals and objectives of these conferences. Additional work to replicate these findings in other environments would provide further evidence of the benefits of this approach.

## Conclusions

Introduction of a standardized, guided template improved the quality of M&M presentations, with medium effect sizes and statistically significant increases in nearly all domains. Standardization of case selection and presentation can promote alignment of the M&M workflow with broader-scope initiatives, departmentally and institutionally. Our template can be implemented broadly across surgical disciplines to improve the quality and effectiveness of these conferences.

## Data Availability

No datasets were generated or analysed during the current study.
